# Metabolic Syndrome Impairs Executive Function in Bipolar Disorder

**DOI:** 10.3389/fnins.2021.717824

**Published:** 2021-08-11

**Authors:** Nina Dalkner, Susanne A. Bengesser, Armin Birner, Frederike T. Fellendorf, Eva Fleischmann, Katja Großschädl, Melanie Lenger, Alexander Maget, Martina Platzer, Robert Queissner, Elena Schönthaler, Adelina Tmava-Berisha, Eva Z. Reininghaus

**Affiliations:** Department of Psychiatry and Psychotherapeutic Medicine, Medical University of Graz, Graz, Austria

**Keywords:** metabolic syndrome, cognition, executive function, bipolar disorder, abdominal obesity

## Abstract

**Background:** Metabolic syndrome (MetS) is more prevalent in individuals with bipolar disorder and has a negative impact on cognition, in particular on executive function, which is already impaired in individuals with bipolar disorder compared to healthy controls.

**Methods:** In a cross-sectional study, we compared 148 euthymic patients with bipolar disorder and 117 healthy controls in cognitive function depending on the diagnosis of MetS. A neuropsychological test battery was used including the Trail Making Test A/B, Stroop Color and Word Interference Test, the d2 Test of Attention Revised, and the California Verbal Learning Test. In addition, MetS variables as well as the defining variables waist circumference, serum triglyceride levels, high-density lipoprotein cholesterol levels, blood pressure, fasting glucose levels, and body mass index were compared between patients and controls. In addition, illness-related variables were associated with MetS in individuals with bipolar disorder.

**Results:** The prevalence of MetS in patients with bipolar disorder was higher than in controls (30.4 vs. 15.4%). Patients with bipolar disorder with MetS had impaired executive function compared to patients without MetS or healthy controls with and without MetS (*p* = 0.020). No MetS effects or interaction MetS × Group was found in attention/processing speed (*p* = 0.883) and verbal learning/memory (*p* = 0.373). Clinical variables (illness duration, suicidality, number of affective episodes, medication, age of onset, and history of psychosis) did not relate to MetS in bipolar disorder (*p* > 0.05).

**Conclusion:** Bipolar disorder comorbid with MetS bears additional risk for impaired executive function. Executive function includes action planning, inhibition, and impulse control and could play a critical role in keeping long-term goals in mind associated with gaining and maintaining a healthy weight.

## Introduction

Bipolar disorder (BD) is a neuropsychiatric illness that occurs in early adulthood (American Psychiatric Association, 2013) and is prevalent in 1–2% of the population (Merikangas et al., 2011; Blanco et al., 2017). It is characterized by recurring episodes of mood changes, featuring recurring episodes of depression and (hypo−)mania. In comparison to the general population, individuals with BD have a decreased life expectancy (Kessing et al., 2015) due to heightened suicide risk, acute psychopathology, sociopsychological consequences (e.g., low socioeconomic status), and psychiatric comorbidities (e.g., substance use disorder), but also due to somatic comorbidities and obesity (Zhao et al., 2016; Yamagata et al., 2017) and related cardiovascular and metabolic illness, such as diabetes mellitus (Crump et al., 2013; Vancampfort et al., 2016) and cardiovascular disease (Westman et al., 2013; Nielsen et al., 2021). Evidently, diseases of metabolic nature constitute particular risk factors for premature mortality in BD (de Almeida et al., 2012).

Metabolic syndrome (MetS) is a cluster of harmful conditions associated with severe health risks and premature mortality, especially in relation to cardiovascular diseases (relative risk (RR), 1.74; 95% confidence interval (CI) (1.29–2.35; Galassi et al., 2006). According to the International Diabetes Federation (IDF), MetS is defined by the co-occurrence of central obesity, raised triglycerides, reduced high-density lipoprotein (HDL), hypertension, and increased fasting blood sugar (Alberti et al., 2006). MetS is a prothrombotic and proinflammatory state characterized by increased inflammatory cytokine activity and increases the risk for atherosclerosis, cardiovascular disease, diabetes mellitus, and chronic kidney disease (McCracken et al., 2018). Prevalence reports of MetS in BD diverge greatly, ranging from 8% (Babić et al., 2010) to 67% (Garcia-Portilla et al., 2008; Grover et al., 2012), with significant differences according to sex, age (Salvi et al., 2011), geographic region, and prescribed medication (Vancampfort et al., 2016). Nevertheless, most studies agree that the prevalence of MetS is significantly higher in individuals with BD than in healthy controls (HC; Czepielewski et al., 2013; Vancampfort et al., 2016; Almasabi et al., 2020), with an odds ratio (OR) as high as 2.94 (95% CI, 2.08–4.17; Almasabi et al., 2020) and that there is an increase in the MetS prevalence among patients with BD in the longitudinal course (Almasabi et al., 2020; Grover et al., 2020). Multiple factors that contribute to these high rates exist, among them psychopharmaceutical effects, inflammatory processes, and unhealthy lifestyle including poor diet and exercise habits (Gurpegui et al., 2012; Swartz and Fagiolini, 2012; Geddes and Miklowitz, 2013). Bai et al. (2016) found the prevalence of MetS to be 29.4% in a sample of 143 individuals with BD. In this study, patients treated with atypical antipsychotics and mood stabilizers (36.3%) as well as atypical antipsychotics alone (36.0%) had a significantly higher prevalence of MetS than those treated with mood stabilizers alone (10.5%, *p* = 0.012). However, the weight-gaining effect of psychotropic medication might not account for obesity in BD alone, since high prevalence rates were observed before the onset of medication commonly used today and are also evident in drug-naïve patients (Maina et al., 2008; Calkin et al., 2009).

Bipolar disorder accompanied by MetS is related to more adverse psychiatric outcomes than BD without MetS; individuals with both BD and MetS were found to have a significantly less favorable response to treatment (McIntyre et al., 2010), poorer global functioning, and more previous hospitalizations (Czepielewski et al., 2013). Accordingly, in obese bipolar individuals, a worse psychiatric outcome (Wildes et al., 2006), more frequent recurrence of illness episodes, a greater number of depressive episodes (*p* < 0.001; Fagiolini et al., 2003), and both a longer duration of depressive episodes (*p* = 0.001; Goldstein et al., 2011) and a greater frequency of comorbid anxiety (*p* < 0.001; Goldstein et al., 2011) than in non-obese individuals were found (Goldstein et al., 2011). However, one study found that these differences were no longer significant after controlling for basic demographic variables (Goldstein et al., 2013). Obesity was further linked to worse functioning and less life satisfaction after half a year of treatment for BD (McElroy et al., 2016). Several researchers have hypothesized these outcomes to be closely tied to neurodegeneration and impaired cognition (Bai et al., 2016; Almasabi et al., 2020).

Cognitive impairment is common among individuals with BD during both euthymia and illness episodes, as confirmed by several meta-analyses (Arts et al., 2008; Bora et al., 2009, 2016; Bourne et al., 2013; Torres et al., 2014; Bortolato et al., 2015; Dickinson et al., 2017; Raucher-Chéné et al., 2017), and is highly related to everyday functioning (*r* = 0.27; 95% CI, 0.22–0.32; *p* < 0.001; Depp et al., 2012). In BD, neuropsychological deficits were particularly found in executive function (*d* > 0.8; Arts et al., 2008), attention (0.5 < *d* < 0.8; Arts et al., 2008), verbal learning and memory (*d* > 0.8; 34; Cipriani et al., 2017; Dickinson et al., 2017), and psychomotor processing speed (*d* = 0.21; Bora, 2018), which is reflected by structural and metabolic changes in the hippocampus, the amygdala, and the prefrontal cortex (de Sá et al., 2016). Cognitive dysfunction may be a vulnerability marker in BD, as it is observed in first-degree relatives of BD patients (Arts et al., 2008; Bora et al., 2009), emerging as early as in premorbid BD stages (Martino et al., 2015). However, it was reported that it does not worsen in the longitudinal course by one meta-analysis (Samamé et al., 2014).

In recent literature, MetS has been linked to cognitive impairments and decline (Siervo et al., 2014). MetS was associated with an increased risk of developing cognitive impairment during a 4-year period in women without psychiatric disorders (OR, 1.66; 95% CI, 1.14–2.41; Yaffe et al., 2009), and MetS was found to increase the risk of progression from mild cognitive impairment to dementia (hazard ratio (HR), 2.69; 95% CI, 1.16–6.27; Atti et al., 2019). In individuals with BD, however, the relationship between MetS and neurocognition has received limited attention (Bora et al., 2019; Restrepo Moreno et al., 2019). A recent systematic review by Bora et al. (2019) reported on three studies comparing cognitive performances of bipolar individuals with and without MetS. Hubenak et al. (2015) found impairments in bipolar patients with MetS in global cognition using a cognitive composite score including values of the Rey Auditory Verbal Learning Test, the Spatial Span and Digit Span from the Wechsler Memory Scale–III, the Continuous Performance Test II, the Tower of London DX, and the Wisconsin Card Sorting Test. Bai et al. (2016) observed impaired executive function using the Wisconsin Card Sorting Test in bipolar individuals with MetS (*p* = 0.0042). Lackner et al. (2016a) reported associations between waist-to-hip ratio (WHR) as well as MetS and impairments in the “Reading the Mind in the Eyes” test, a theory of mind task (*p* < 0.05).

In line with this, there are more studies dealing with obesity effects on cognition in BD. A modest but significant relationship between obesity and cognitive deficits in BD was reported by Bora et al. (2019) (*d* = 0.36), with most robust differences between obese/overweight vs. normal-weight patients with BD in measures of executive functions (*d* = 0.61) and processing speed (*d* = 0.48). In earlier studies, obesity and abdominal obesity have been linked to cognitive impairment in euthymic BD patients (Yim et al., 2012; Lackner et al., 2016b). Yim et al. (2012) found negative correlations between body mass index (BMI) and psychomotor processing speed in patients with BD (*r* = −0.32, *p* < 0.001), and overweight patients had lower executive function (*p* = 0.013) than normal-weight subjects. In preliminary data of the BIPFAT sample, our study group demonstrated associations between waist-to-height ratio (WTHR) and measures of attention and psychomotor processing speed, verbal learning and memory, and executive function in euthymic bipolar individuals (Lackner et al., 2016b). Mora et al. (2017) found impairments in a global cognitive index (including executive function, inhibition, attention, and processing speed) in euthymic overweight (*p* = 0.018) and obese bipolar individuals (*p* = 0.033) in a 6-year follow-up period. Accordingly, our study group presented preliminary data of the BIPLONG study, indicating that high BMI predicted decline in working memory (β = −0.42, *p* = 0.008; 58). In addition, Bond et al. (2017) found that low cognitive functioning predicted weight gain in BD (β = −0.273, *p* = 0.039). Furthermore, obesity contributes to the decrease in cognitive functioning, markedly affecting processing speed (*p* < 0.01) as well as reasoning and problem solving (*p* < 0.05; La Montagna et al., 2017). Albeit research on the influence of both obesity and MetS on cognition in BD points to a reciprocal association, studies remain scarce.

This study is filling a gap in the literature by investigating the combination of BD on MetS on cognitive dysfunction in domains that have not previously been examined, hypothesizing that individuals with BD and additional MetS show more cognitive deficits in subdomains compared to patients with BD without MetS. The strengths of this study are (1) the well-characterized sample of euthymic individuals with a BD diagnosis according to the “Diagnostic and Statistical Manual of Mental Disorders, Fourth Edition” (DSM-IV; Wittchen et al., 1997), (2) the use of strict IDF criteria to assess MetS, (3) the use of a comprehensive neuropsychological test battery including three cognitive domain scores: attention/processing speed, verbal learning/memory, and executive function, and (4) the inclusion of an HC group. To add to existing literature, we were also interested in the impact of MetS on BD illness variables.

## Materials and Methods

### Participants

The study was conducted at the research unit and outpatient center for BD at the Medical University Graz, Department of Psychiatry and Psychotherapeutic Medicine. This investigation was part of the ongoing BIPFAT/BIPLONG study that seeks to determine complete actual and lifetime psychiatric history in association with lipid metabolism, lifestyle, brain function, and cognition in BD in the longitudinal setting. This includes the assessment of psychiatric symptomatology, somatic and psychiatric comorbidities, fasting blood samples, anthropometric measurements, cognitive tests, electroencephalogram, magnetic resonance imagining of the brain, and questionnaires on mood and lifestyle. For study design and preliminary results, see our previous reports (Reininghaus et al., 2014; Dalkner et al., 2021). The patient inclusion criteria were diagnosis of BD using the structured clinical interview for DSM-IV (Wittchen et al., 1997), age between 18 and 70 years, and premorbid IQ ≥ 80. The exclusion criteria were any diagnosis of a lifetime history of schizophrenia or dementia. For the current analysis, the BIPFAT/BIPLONG data from the first test point were used, and patients with a complete data set and a score on the Hamilton Depression Scale (HAMD; Hamilton, 1967) ≤10 and a Young Mania Rating Scale (YMRS; Young et al., 1978) score ≤11 were included. For HC, exclusion criteria were lifetime history of a psychiatric disorder or first-degree relatives with severe psychiatric disorders (BD, schizophrenia, or major depression). All participants provided written informed consent prior to participation in the study. The study was approved by the local ethics committee in accordance with the Declaration of Helsinki (EK number: 24–123 ex 11/12).

### Measurement

Anthropometric and fasting biochemical assessments were performed to investigate MetS prevalence according to the IDF criteria (Galassi et al., 2006), including central obesity (a waist circumference of >94 cm in men or >80 cm in women) in addition to any two of the following criteria: (a) fasting serum triglyceride levels of ≥150 mg/dl, (b) fasting HDL cholesterol levels of <40 mg/dl in men or <50 mg/dl in women, (c) blood pressure of ≥130/85 mmHg, or (d) a fasting glucose level of ≥100 mg/dl. Patients receiving medication for hypertension, diabetes mellitus, or hyperlipidemia were considered to fulfill the MetS components criteria too. BMI [kg/m^2^] was categorized as underweight (under 18.5), normal weight (18.5–24.9), overweight (25–29.9), and obese (over 30; Nuttall, 2015).

In addition, anamnestic data and psychiatric history (BD type I/type II, illness duration, history of suicidal behavior, history of psychosis, number of affective episodes, age of onset, and mood stabilizing medication) were assessed by trained research staff. Psychosocial functioning was evaluated with the German version of the Global Assessment of Functioning (Wittchen et al., 1997).

#### Neuropsychological Assessment

##### Attention and processing speed

The Trail Making Test part A (TMT-A; Reitan, 1958) measures individual attention and psychomotor speed and was included in this domain. Additionally, the word- and color-naming trials from the Color and Word Interference Test by J. R. Stroop (Bäumler, 1985) were included in this domain, as these measures often emerge under “processing speed” factors in factor analytic neuropsychological studies in BD (Langenecker et al., 2010; Ryan et al., 2012). The revised version of the d2 Test of Attention Revised (d2-R) by Brickenkamp et al. (2010) measures the individual attention and concentration performance and accuracy while differentiating similar visual stimuli (Brickenkamp, 2002).

##### Verbal memory

To assess verbal learning and memory, the German version of the California Verbal Learning Test (CVLT) by Niemann et al. (2008) was used, including the recall trials 1–5, the CVLT short delay free, the CVLT short-cued recall, the CVLT long delay free, and the CVLT long delay cued recall into the verbal memory domain score.

##### Executive function

The measures included in the executive function domain include the TMT-B (Reitan, 1958) and the interference trial from the Color and Word Interference Test by J. R. Stroop (Bäumler, 1985).

### Statistical Methods

We compared patients with BD and HC (with and without MetS) with regard to demographic and metabolic variables with *F*-tests for continuous variables and chi-square tests (χ^2^) for categorical ones. The differences in cognitive raw scores and MetS variables were planned to test using two-way analyses of covariance (ANCOVAS) with grouping variable Group (BD vs. HV) and MetS (with vs. without) controlling for age, sex, and education. However, as relevant assumptions for statistical tests were violated for the metabolic variables and cognitive raw scores, Mann–Whitney *U*-tests were applied. Differences in other clinical variables (history of suicidality, number of affective episodes, history of psychosis, illness duration, age of onset, and global functioning) between patients with MetS and patients without MetS in the BD group were compared with Mann–Whitney *U*-tests for non-parametric data and chi-square tests (χ^2^).

For each primary cognitive measure, the subjects’ raw scores were converted into z-scores and then summed up into three cognitive domain z-scores: (a) attention/processing speed (TMT-A, Stroop color word reading, Stroop color naming, and d2-R), (b) executive function (TMT-B and Stroop interference), and (c) verbal learning/memory (CVLT trial 1–5, CVLT short delay free recall, CVLT short delay cued recall, CVLT long delay free recall, and CVLT long delay cued recall), which served as primary neuropsychological outcome variables. On measures of reaction time (on which high scores indicate low performance), z-scores were reversed before forming the domain score.

A two-way multivariate analyses of covariance (MANCOVA) with Group (BD vs. HC) and MetS (with vs. without) as independent variables; the cognitive domain scores (attention/processing speed, verbal learning/memory, and executive function) as dependent variables; and age, sex, education, and smoking as covariables were performed followed by univariate tests or each of the domain scores. MANCOVA key assumptions (linearity, normality, and homogeneity) were verified graphically and with the Kolmogorov–Smirnov test and Levene’s test. The assumption of homogeneity was violated for the domains memory and executive function in the Levene’s test (*p* < 0.001); therefore, the Hotelling T2 test, which is suitable for heteroscedastic two-way MANOVAs, was computed (Zhang, 2011).

## Results

In sum, data of 265 participants were included (148 participants with BD and 117 HC). The BD group consisted of 82 males and 66 females, who participated with an average age of 42.41 ± 12.52 years (min = 18 years, max = 68 years). In the HC group, 44 males and 73 females were included, with a mean age of 35.79 ± 14.29 years (min = 19 years, max = 69 years).

Table 1 presents the differences between individuals with BD and HC in demographics, metabolic parameters, and the cognitive raw scores with and without MetS. The results showed that the prevalence of MetS was 30.4% among patients with BD and 15.4% in HC. Women and men differed in MetS rates in the BD group (χ^2^(1) = 10.63, *p* = 0.001) comprising 34 men and 11 women with MetS, but not in HC (χ^2^(1) = 2.92, *p* = 0.087) including 10 men and 8 women with MetS. In patients with BD, more males and more smokers were found than in HC. In addition, in the BD group, higher IQ/HAMD/YMRS scores, higher BMI, higher waist circumference, elevated triglycerides, lower HDL levels, and higher glucose levels were found. In individuals with MetS, higher values in the metabolic variables and lower cognitive test performance (in cognitive raw scores) were found. In Table 2, the clinical characteristics of bipolar patients with MetS and without MetS are listed. No differences between patients with and without MetS were found in medication or in any other clinical variables.

**TABLE 1:** Baseline demographic and clinical variables for participants in the total sample (BD vs. HC) and by MetS group.

	**Total sample**	**BD**		**HC**			
		**With MetS**	**Without MetS**	**With MetS**	**Without MetS**		

	***n* = 265 M (±SD)**	***n* = 45 M (±SD)**	***n* = 103 M (±SD)**	***n* = 18 M (±SD)**	***n* = 99 M (±SD)**	***Differences between* BD vs. HC F/χ^2^/U**	***Differences between MetS* vs. *without MetS* F/χ^2^**
Age (years)	39.49 (13.71)	44.16 (12.59)	41.65 (12.48)	45.80 (13.56)	33.97 (13.72)	2.20	12.38**
Sex (n, %)							
Male	126 (47.5%)	34 (41.5%)	48 (58.5%)	10 (22.7%)	34 (77.3%)	8.30*	16.47**
Female	139 (52.5%)	11 (16.7%)	55 (83.3%)	8 (11.0%)	65 (89.0%)		
Smoking (n, %)	90 (34.0%)	23 (43.7%)	45 (51.1%)	6 (33.3%)	16 (16.2%)	21.46***	5.37*
IQ	111 (14)	105.58 (15.24)	110.29 (13.23)	120.61 (14.73)	112.41 (13.84)	6897.00*** *z* = −2.773	5693.50 *z* = −1.211
HAMD	3 (3)	4.29 (2.98)	3.87 (3.27)	0.54 (1.45)	0.30 (0.86)	1783.00*** *z* = −9.574	3963.00*** *z* = −2.618
YMRS	1 (2)	1.49 (2.77)	1.20 (2.26)	0.00 (0.00)	1.49 (2.77)	4436.00*** *z* = −5.198	4788.00 *z* = −0.951
BMI	26.31 (5.82)	32.47 (7.49)	25.97 (4.45)	30.65 (4.08)	23.09 (3.34)	5245.50*** *z* = −5.508	1789.50*** *z* = −8.611

**MetS variables**							

Waist circumference (cm)	88.51 (15.97)	107.24 (18.84)	88.14 (13.02)	104.22 (8.65)	81.15 (11.51)	3241.50*** *z* = −4.328	691.00*** *z* = −8.041
Triglyceride (mg/dl)	115 (77)	200.59 (110.71)	113.13 (66.87)	154.06 (82.44)	77.71 (41.17)	5063.00*** *z* = −5.395	2032.50*** *z* = −7.946
HDL cholesterol	62 (20)	45.72 (13.65)	60.70 (16.79)	48.78 (15.98)	70.54 (19.47)	5825.50*** *z* = −4.054	2355.00*** *z* = −7.203
Systolic blood pressure (mmHg)	125 (15)	135.36 (17.02)	122.10 (13.92)	137.94 (13.33)	119.76 (14.16)	6795.50 *z* = −1.742	2444.50*** *z* = −6.825
Diastolic blood pressure (mmHg)	83 (10)	88.80 (8.88)	82.14 (9.00)	94.22 (8.24)	80.00 (9.98)	7180.50 *z* = −0.981	2829.00*** *z* = −6.019
Glucose (microg/dl)	93 (17)	107.05 (29.04)	89.48 (10.39)	103.72 (16.04)	87.82 (8.30)	6487.00* *z* = −2.544	2360.00*** *z* = −7.128

**Cognitive variables**							

TMT-A	31.78 (13.60)	39.55 (18.83)	34.42 (11.39)	29.88 (7.59)	25.85 (11.08)	4810.00*** *z* = −6.211	4763.50*** *z* = −3.012
TMT-B	71.51 (36.86)	97.16 (58.85)	77.00 (31.31)	65.03 (22.85)	55.31 (19.67)	4713.00*** *z* = −6.368	4659.00*** *z* = −3.208
d2-R	164.47 (53.05)	136.40 (46.17)	146.32 (43.51)	173.78 (38.51)	194.43 (52.60)	3983.50*** *z* = −7.545	4851.00*** *z* = −2.847
Stroop color word reading	30.38 (5.50)	32.59 (6.53)	31.33 (5.52)	29.79 (3.74)	28.50 (4.61)	5980.50*** *z* = −4.322	5090.50* *z* = −2.396
Stroop color naming	46.80 (8.54)	50.72 (9.05)	48.73 (8.98)	43.87 (4.69)	43.55 (6.99)	5335.50*** *z* = −5.363	5150.50* *z* = −2.283
Stroop interference	75.38 (21.08)	89.29 (29.40)	79.27 (20.78)	67.43 (10.39)	66.45 (12.01)	4832.00*** *z* = −6.176	4795.00*** *z* = −2.952
CVLT trial 1−5	56 (13)	49.78 (12.79)	52.86 (12.82)	52.00 (13.69)	62.43 (9.12)	5099.00*** *z* = −5.747	4242.50*** *z* = −3.994
CVLT short delay free recall	12 (3)	10.36 (3.69)	10.53 (3.39)	11.17 (4.12)	13.20 (2.52)	5067.50*** *z* = −5.825	5168.00* *z* = −2.261
CVLT short delay cued recall	12 (3)	10.96 (3.31)	11.48 (3.07)	11.83 (3.37)	13.76 (2.19)	5035.50*** *z* = −5.887	4769.50*** *z* = −3.021
CVLT long delay free recall	12 (3)	10.96 (3.59)	11.28 (3.47)	11.39 (4.39)	13.68 (2.54)	5369.50*** *z* = −5.349	4990.00*** *z* = −2.605
CVLT long delay cued recall	13 (3)	11.07 (3.44)	11.93 (3.09)	11.56 (4.02)	14.00 (2.18)	5379.00*** *z* = −5.344	4511.00*** *z* = −3.521

Results from univariate ANCOVAs, Mann–Whitney *U*-tests, or chi-square tests. ***p < 0.001; **p < 0.01; *p < 0.05.

**TABLE 2:** Clinical characteristics in participants with BD with and without MetS.

	**BD with MetS**	**BD without MetS**	***Differences between the MetS groups***
	*n* = 45 M (±SD)	*n* = 103 M (±SD)	T/χ^2^/U
Subtype of BD type 1/type II (*n*)	32/13	68/33	0.21
Duration of BD	18.50 (13.37)	19.04 (11.25)	1872.50 *z* = −0.608
History of suicide thoughts *(n)*	41	93	−1.21
Number of suicide attempts	0.36 (0.63)	0.45 (0.60)	1577.00 *z* = −0.971
History of psychosis (*n*)	23	62	0.75
Number of depressive episodes	12.05 (15.23)	11.58 (13.52)	1622.00 *z* = −0.589
Number of manic episodes	7.39 (9.31)	7.58 (12.20)	1764.00 *z* = −0.305
Age at onset of BD	25.50 (12.08)	23.48 (9.13)	1936.00 *z* = −0.604
Global assessment of functioning	67.83 (14.16)	70.78 (12.09)	1.23
Treatment with Lithium (*n*)	15	30	0.42
Treatment with Atypical Antipsychotics (*n*)	18	55	2.31
Treatment with Antiepileptics (*n*)	8	23	0.29
Mood stabilizer combination (*n*)	9	28	0.72

*Results from chi-square tests and Mann–Whitney *U*-tests.*

**FIGURE 1:**
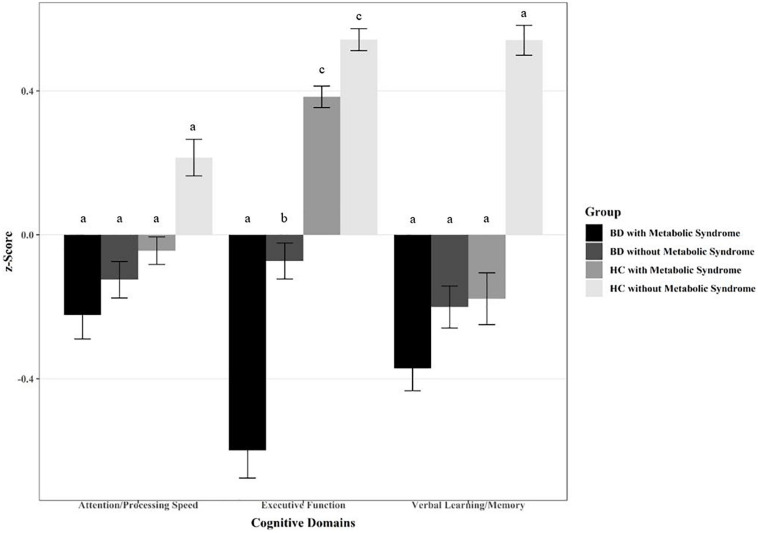
Differences in cognitive domains in individuals with bipolar disorder (BD) with and without metabolic syndrome (MetS) in comparison with healthy controls (HC) with and without MetS. Different letters indicate significant mean differences at *p* < 0.05.

The MANCOVA comparing patients with BD with HC (factor Group) and on the presence of MetS (factor MetS) in three cognitive domains found a significant effect of Group [*F*(1,253) = 10.76, *p* < 0.001, η_*p*_^2^ = 0.11] and a significant interaction MetS × Group [*F*(1,251) = 4.08, *p* = 0.007, η_*p*_^2^ = 0.05]. The factor MetS was not significant [*F*(1,251) = 0.73, *p* = 0.533, η_*p*_^2^ = 0.01]. Age [*F*(1,251) = 15.52, *p* < 0.001, η_*p*_^2^ = 0.16], sex [*F*(1,251) = 8.68, *p* < 0.001, η_*p*_^2^ = 0.09], and education (*F*(1,251) = 7.04, *p* < 0.001, η_*p*_^2^ = 0.08) were significant confounding variables. Smoking had no significant confounding effect [*F*(1,253) = 0.10, *p* = 0.960 η_*p*_^2^ = 0.001]. The univariate results showed that groups (BD vs. HC) differed in the domain executive function (see Table 3). In addition, a significant interaction MetS × Group in executive function was observed. Patients with BD had poorer performance in executive function than HC, and patients with BD and additional MetS had the poorest performance (see Figure 1). For the interactions, scatterplots with the regression lines are shown in the Supplementary Material. Table 3 gives the univariate test statistics following MANCOVA. *Post hoc t*-tests indicated that within the BD group, patients with MetS had a lower score in executive function than patients without MetS (*t*(146) = 2.59, *p* = 0.012). In the HC group, there was no difference in executive function in dependence to MetS (*t*(115) = 1.26, *p* = 0.212).

**TABLE 3:** Univariate results following two-way MANCOVA.

	**Main effect Group**			**Main effect MetS**			**Interaction Group × MetS**		
	***F***	***p***	**η*_*p*_^2^***	**F**	**p**	**η*_*p*_^2^***	**F**	**p**	**η*_*p*_^2^***
**Cognitive domain scores**									
Attention/processing speed	2.11	0.148	0.01	0.13	0.722	0.00	0.02	0.883	0.00
Verbal learning/memory	3.80	0.052	0.02	0.80	0.373	0.00	2.43	0.120	0.01
Executive function	**30.80**	**<0.001**	**0.11**	1.96	0.163	0.01	**5.47**	**0.020**	**0.02**

*Univariate results following two-way MANCOVA with Group (BD vs. HC) and MetS (with vs. without) as independent variables, the cognitive domain scores (attention/processing speed, verbal learning/memory, and executive function) as dependent variables, and controlling for age, sex, education, and smoking. Significant results (*p* < 0.05) are presented in bold letters.*

## Discussion

In accordance with previous studies, we found that MetS cases were higher in the bipolar group (30%) than in the control group (15%; Garcia-Portilla et al., 2008; Babić et al., 2010; Salvi et al., 2011; Grover et al., 2012, 2020; Czepielewski et al., 2013; Vancampfort et al., 2015; Almasabi et al., 2020). In HC, the prevalence was lower than in an Austrian comparative group (Wascher, 2002), possibly caused by younger age and higher educational level in the control group. Therefore, age, education, and sex were included in most analyses as control variables. Individuals with BD and MetS had impaired executive function in comparison to patients without MetS as well as HC with and without MetS. For attention/processing speed and verbal learning/memory, no significant interactions of MetS and BD were found.

Interestingly, no MetS effects were observed in the MANCOVA to test differences in cognitive domain scores across all individuals. However, negative z-scores were observed in individuals with MetS in the domains attention/processing speed and more obvious in verbal learning/memory. Moreover, MetS effects were found in the raw cognitive domain scores independent from psychiatric diagnosis. This finding was in line with previous studies indicating that MetS affects global cognition in individuals with psychiatric disorders (Hubenak et al., 2015) as well as in HC (Yaffe et al., 2009; Siervo et al., 2014; Atti et al., 2019). However, as mostly non-parametric tests were used for raw scores, these results were not controlled for relevant covariables. Nevertheless, our findings suggest that MetS is not a favorable factor for cognitive function.

In sum, the current results supported our hypothesis that patients with BD and comorbid MetS showed impaired executive function in comparison to patients without MetS and HC. Our results were in line with other studies investigating the effects of MetS and obesity on cognition (Hubenak et al., 2015; Bai et al., 2016; Bora et al., 2019). In the association between obesity and cognition, Bora et al. reported most robust effects (*d* = 0.61) in the subdomain executive function (Bora et al., 2019). Accordingly, Bai et al. observed impairments in bipolar individuals with MetS in the Wisconsin Card Sorting Test, measuring executive function as well (Bai et al., 2016). Hubenak et al. (2015) calculated a global cognition score including measures of executive function as well (e.g., Tower of London, Wisconsin Card Sorting Test). Recently, elevated triglyceride levels, as one important MetS factor, could be linked to worse cognitive flexibility and set-shifting performance in individuals with BD but not in controls (Naiberg et al., 2016; Van Rheenen et al., 2021). In future studies, the impact of the single MetS variables on cognitive test performance should be investigated too. A meta-analysis including 72 studies to investigate the effects of obesity on executive function in otherwise healthy individuals found that obese participants showed broad impairments only in inhibition and working memory; no differences between overweight and normal-weight individuals emerged on tasks assessing cognitive flexibility, decision making, and verbal fluency (Yang et al., 2018). Executive function is an umbrella term and refers to a set of cognitive skills needed for self-control, action planning, and managing behaviors. In addition, executive function includes working memory, mental flexibility, inhibition, and impulse control. Such skills enable people to follow directions, focus, control emotions, and attain goals (Carlson et al., 2013). From resisting temptation to keeping long-term goals in mind, such functions are needed to maintain healthy weight and lifestyle, including regular dinner intakes and physical activity (Tee et al., 2018; Serra et al., 2020). Thus, executive function has an obvious relevance to overweight and metabolic risk and plays a critical role in the everyday lives of individuals with BD.

It should be noted that in the current study, executive function was assessed with two tests. The first, TMT-B, is a well-established test to evaluate cognitive flexibility and ability to maintain a complex response, although researchers argue which of these abilities contributes more to TMT-B performance (Kortte et al., 2002). In addition, the TMT-B was found to involve working memory as well (Sánchez-Cubillo et al., 2009; Llinàs-Reglà et al., 2017). The overlap of the functions necessary to complete the TMT-B has led to researchers using this test to assess both functions, coming to different conclusions about the cognitive abilities of individuals with BD (Lima et al., 2018). The second tool for assessment was the interference task of the Stroop’s Color and Word Interference Test, which measures cognitive inhibition, the ability to focus one’s attention selectively (Diamond, 2013). However, the concept of inhibition was criticized as being composed of several ideas instead of a single one (MacLeod et al., 2003), increasing the difficulty of measuring inhibition. The score of this test could be predicted by both conflict monitoring and the speed of visual search (Periáñez et al., 2021).

Metabolic syndrome effects or interaction MetS × Group were shown for neither attention/processing speed nor verbal learning/memory. Likewise, no differences in clinical outcome variables (subtype of BD, illness duration, history of suicidality, history of psychosis, number of affective episodes, age of onset, and mood stabilizing medication) between patients with MetS and without MetS could be observed. This finding was in contrast to other studies demonstrating negative effects of MetS on the outcome of BD/depression (McIntyre et al., 2010; Bai et al., 2016). Therefore, future longitudinal studies are needed to explore the complex interplay between clinical outcome variables and MetS.

Current literature indicates that beside polygenetic predisposition, neurotransmitter dysbalances, and psychosocial burdens, inflammatory process alterations are highly important in the etiopathogenesis of BD (Leboyer et al., 2012). Chronic low-grade inflammation with the activation of proinflammatory cytokines in acute illness episodes, but also in euthymia, is discussed as one of the most important pathophysiological underpinnings (Anderson and Maes, 2015; Bai et al., 2015). These inflammatory processes are closely linked to changes in the pathways of tryptophan, serotonin, and melatonin. Additionally, chronic proinflammatory and oxidative processes have been identified to be involved in the degeneration of the central nervous system in BD, such as neural activity and brain structure, which in turn is related to cognitive decline (Anderson and Maes, 2015). These mechanisms impair especially executive function (Tseng et al., 2021), and additionally, a link to reduced frontal cortex volume was found (Chen et al., 2020). Interestingly, the inflammatory process of MetS is suspected to share a common biological pathway with BD: the kynurenine pathway, which stimulates the production of neurotoxic metabolites as a response to stress, thus contributing to endothelial dysfunction (Halaris, 2017; Benedetti et al., 2020). Furthermore, inflammatory cytokines may trigger the hypothalamic–pituitary–adrenal axis, contributing to a heightened stress reaction (Rosenblat and McIntyre, 2017). Markers of inflammation are generally associated with poorer cross-sectional cognitive function and faster longitudinal decline in various domains of cognition (Beydoun et al., 2018). Regarding these findings, inflammation processes of both MetS and BD possibly reinforce each other, contributing to a greater decline in cognitive function.

In view of the problems arising from the combination of BD and MetS, it is integral to help these individuals implement appropriate changes in lifestyle. Overweight, hypertension, chronic low-grade inflammation, and lipid parameters can be influenced by diet and evidence-based nutritional and pharmacological interventions (Custodero et al., 2018). In addition, regular physical activity stabilizes the cytokine production and reduces the systemic proinflammatory state (Alsuwaidan et al., 2009; Rosa et al., 2011). A better performance in cognition was shown in female individuals conducting vigorous physical activity compared to moderate or low activity (Fellendorf et al., 2017). Influencing the inflammatory system beneficially by having a healthier lifestyle could therefore not only positively impact weight and MetS symptoms but also cognitive functioning.

## Limitations

The major strengths of this study were the investigation of a euthymic BD sample and the inclusion of an HC group. However, several limiting factors were found: as this study had a cross-sectional design, causality could not be determined. In addition, it was not feasible to take participants’ type of medication into account. The major limitation is that we have included data from patients receiving medication for hypertension, diabetes mellitus, and/or hyperlipidemia who were considered to fulfill the MetS components criteria too. This obviously has led to a heterogeneous group, which could bias interpretation. Although there was no significant difference in mood stabilizing medication in dependence of MetS, future studies should take other medications and their possible effects/interactions more into account. In addition, relative impacts of MetS variables were not explored, which may elucidate aspects of MetS with greater contribution to executive dysfunction in this population.

## Implications

Our findings add to a growing body of literature that suggests that the prevalence of MetS in BD is high, which requires regular monitoring and adequate prevention and treatment of cardio-metabolic risk factors (Baillot et al., 2017; Tully et al., 2018). In addition, the prevention of abdominal obesity and prevention of lifestyle changes (including healthy diet and exercise) should become firm components in treatment programs for BD. Moreover, individuals with BD should receive information about the harmful consequences of overweight and MetS on cognitive parameters, which in turn impair quality of life and illness outcome (Depp et al., 2012).

## Conclusion

This study has a unique contribution by investigating the combination of BD on MetS on cognitive dysfunction in domains that have not previously been examined. Patients with BD and comorbid MetS have broad impairments in the domain of executive function compared to those patients without MetS and HC. Monitoring of MetS risk factors, such as nutrition, exercise, alcohol intake, and nicotine intake, is important not only for physical health but also for cognitive outcomes. In conclusion, MetS is a severe risk factor for executive function deficit in euthymic individuals with BD.

## Data Availability Statement

The raw data supporting the conclusions of this article will be made available by the authors, without undue reservation.

## Ethics Statement

The studies involving human participants were reviewed and approved by the Ethikkommission der Medizinischen Universität Graz, Auenbruggerplatz 2, 1. OG, 8036 Graz. The patients/participants provided their written informed consent to participate in this study.

## Author Contributions

ND was responsible for the study conception, patient recruitment, data collection, testing, statistical analyses, coordination, writing of the first draft, and publication of data. SB, AB, FF, ML, AM, MP, and RQ was responsible for data collection, testing, revised, and edited the manuscript. EF collected literature, helped with writing, revised, and edited the manuscript. KG and AT-B did revision for important intellectual content. ES helped with interpretation of the data, created the figure, and did revision for important intellectual content of the manuscript. ER was involved in the conception of the study, supervised, and guided through the whole process of analysis and publication. All authors edited and approved the final manuscript.

## Conflict of Interest

The authors declare that the research was conducted in the absence of any commercial or financial relationships that could be construed as a potential conflict of interest.

## Publisher’s Note

All claims expressed in this article are solely those of the authors and do not necessarily represent those of their affiliated organizations, or those of the publisher, the editors and the reviewers. Any product that may be evaluated in this article, or claim that may be made by its manufacturer, is not guaranteed or endorsed by the publisher.
