# Supplementary figures and images for: Metabolic Syndrome Impairs Executive Function in Bipolar Disorder
Source: Front Neurosci. 2021 Aug 11;15:717824. doi: 10.3389/fnins.2021.717824 (PMC8385126; doi:10.3389/fnins.2021.717824)

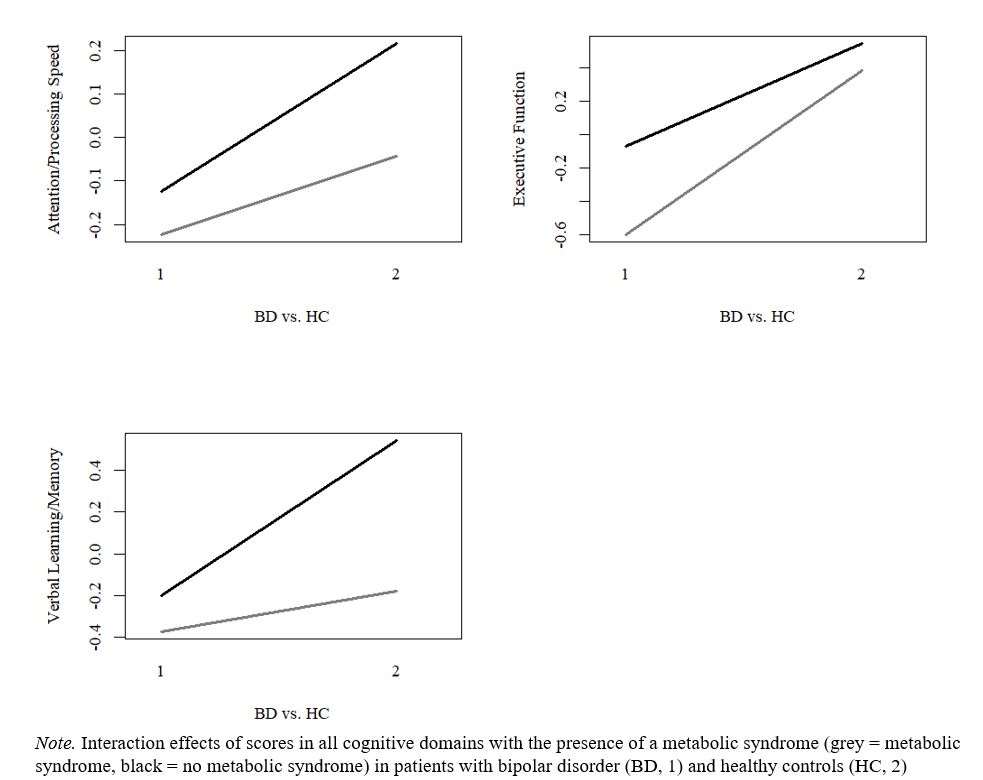

Supplement: Supplementary file 1 [file Image_1.JPEG]
